# Validity and reliability of a dish-based semi-quantitative food frequency questionnaire for assessment of energy and nutrient intake among Iranian adults

**DOI:** 10.1186/s13104-020-04944-3

**Published:** 2020-02-24

**Authors:** Azam Doustmohammadian, Maryam Amini, Ahmad Esmaillzadeh, Nasrin Omidvar, Mitra Abtahi, Monireh Dadkhah-Piraghaj, Bahareh Nikooyeh, Tirang R. Neyestani

**Affiliations:** 1 Department of Nutrition Research, Faculty of Nutrition and Food Technology, National Nutrition and Food Technology Research Institute, Shahid Beheshti University of Medical Sciences, No. 7., Hafezi St., Farahzadi Blvd., P.O.Box: 19395-4741, Tehran, 1981619573 Iran; 2 Department of Community Nutrition, School of Nutritional Sciences and Dietetics, Tehran University of Medical Sciences, Tehran, Iran; 3 Department of Community Nutrition, Faculty of Nutrition and Food Technology, National Nutrition and Food Technology Research Institute, Shahid Beheshti University of Medical Sciences, Tehran, Iran

**Keywords:** Validity, Reliability, Dish-based, Food frequency questionnaire, FFQ

## Abstract

**Objective:**

This study aimed to assess the validity and reliability of a dish-based, semi-quantitative food frequency questionnaire (DFFQ) for epidemiological studies in Iran. The DFFQ included 142 items (84 foods and 58 mixed dishes) which was filled in by 230 adults (110 men). All participants completed two separate DFFQs with a 6 months interval as well as six 24-h recalls, each month. Dietary biomarkers and anthropometric measurements were made. The validity was evaluated by comparing the DFFQ against 24-h dietary recalls and dietary biomarkers, including serum retinol and beta-carotene. Reliability was evaluated using intra-class correlation coefficient (ICC) and validity was determined by unadjusted and energy adjusted correlation coefficients (CC), de-attenuated CC, and cross-classification analyses.

**Results:**

ICC for reliability ranged between 0.42 and 0.76. De-attenuated CC for the FFQ and the 24-h recalls ranged between 0.13 and 0.54 (Mean = 0.38). The de-attenuated CC between the DFFQ and plasma levels of retinol and beta-carotene were 0.58 (P = 0.0001) and 0.40 (P = 0.0001), respectively. Cross-classification analysis revealed that on average 73% were correctly classified into same or adjacent quartiles and 5% were classified in opposite quartiles.

## Introduction

Dietary factors play an important role in development of non-communicable diseases (NCDs) [1]. To have a better understanding of any relationship between diet and NCDs, evaluation of habitual intake over a long period of time is needed [2]. Assessment of dietary habits is crucial in large, prospective epidemiological studies which provide a clear understanding of health status. Furthermore, having reliable dietary data allows researchers to examine the relationship between food and nutrients consumption and the susceptibility to diseases [3]. However, since eating behavior is a cultural issue [4, 5] and a dietary assessment tool developed for one population may not be appropriate for the other.

Food frequency questionnaires (FFQs) are widely used to assess dietary patterns in different societies. FFQs, especially food-based FFQs (FFFQs), are reliable tools which can be easily used in countries whose dietary patterns are based on single foods rather than mixed dishes [6–8]. Dish-based FFQs (DFFQ) may have several advantages over FFFQs in the societies whose dietary practices are different from western countries. It is documented that compared to FFFQs a DFFQ calculated antioxidant vitamins, phytochemicals and fatty acids more accurately [3].

Furthermore, a DFFQ can, facilitate evaluation of dietary intake over a long period, since it is much easier to recall the frequency intake of mixed dishes rather than individual ingredients included in them [9].

A number of questionnaires, mainly FFFQ, have been developed in both Western countries [6–8, 10–12] and Asian countries [13–16], including Iran [4, 17–19]. However, Iranian dietary pattern is typically characterized by a plenty of mixed dishes. The dishes contain several ingredients which are cooked by different methods.

Therefore, it is very challenging, if not impossible, to estimate usual intake of single ingredients through an FFFQ. Several international studies have developed DFFQs for epidemiological studies [3, 16, 20]. However, there were some limitations namely not considering seasonal variations of foods [3], the generalizability [16, 20], and the gold standards affairs which have limited their usage as a valid tool. To the best of our knowledge, no earlier study in Iran has developed a validated DFFQ. Despite presence of two formerly developed DFFQs in Iran their applicability are limited due to validity issues or the specific target groups for which the FFQs were designed [21, 22].

The purpose of the current study was to evaluate the validity and reliability of a novel dish-based, semi-quantitative FFQ [23] to be used for epidemiological studies in Iran

## Main text

### Methods

#### Study design and participants

A total of 230 male and female adults whose ages ranged between 18 and 65 years, were non-smokers (due to probable effect of smoking on biomarkers level), non-pregnant and/or nursing, living in Tehran, and were not on a specific diet were recruited in the study. Samples size was calculated based on Willett’s recommendation. According to him 100 to 200 participants seems to be a reasonable sample size for such a validation study [24]. We supposed five age subgroups including 18 to 30, 31 to 40, and 41 to 50 years old with 50 participants in each of them and 51-years old to higher with 75 participants. Details of sampling process is presented in Additional file 1: Figure S1. The convenience sample consisted of volunteers of neighboring banks, staff of NNFTRI, Taxi Organization of Tehran, a Local Community in district 21 of Tehran and ordinary people as well as university students who were invited directly in their workplaces or were informed by the participants of the study. All participants were paid cash donations for their contribution to the study.

#### Food frequency questionnaire (FFQ)

**Fig. 1:**
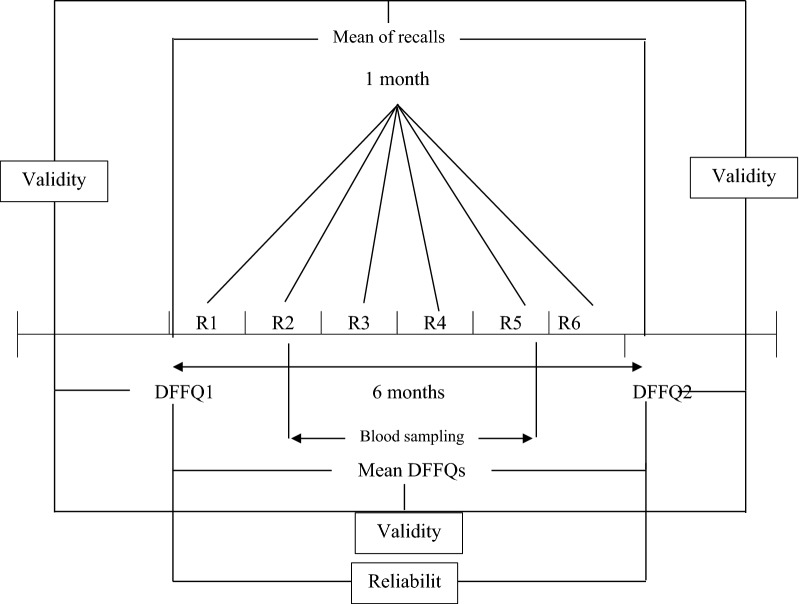
Study design. R = 24-h dietary recall, DFFQ = Dish-based food frequency questionnaire. DFFQ1 and DFFQ2 were completed 1 month before the first recall (winter 2016) and 1 month after the 6th recall (summer of 2016), respectively; six 24-h dietary recalls were collected on a consecutive monthly basis

To evaluate validity and reliability of the FFQ several steps were taken. Summary of steps taken for the validation of the DFFQ are presented in Fig. 1.

I-Development of the DFFQ

A previously developed dish-based semi-quantitative FFQ was used to assess monthly subjects’ habitual dietary intake [25]. The 142 items questionnaire included 84 food items and 58 mixed dishes and was completed by trained interviewers through face to face interview with the participants. Visual aids including locally used utensils were provided to help the participants to understand the concept of the portion sizes. All subjects were supposed to complete 2 individual FFQs with a 6 month interval, one in winter and another in summer of 2016.

II-Criterion validation

In this section, data from the FFQ was evaluated against six 24-h recalls and dietary biomarkers.

II-I-24-h dietary recall

Six 24-h recalls were collected as a reference method almost every month. Multiple probing questions were used to complete each 24-h dietary recall including diverse portion size descriptions, food models and detailed food preparation and cooking methods by interviewing the person responsible for cooking.

II–II-Dietary biomarkers

Five milliliter of fasting antecubital venous blood was taken from all participants. After keeping at room temperature (RT) for 20–30 min at dark, blood samples were centrifuged at 800*g* for 20 min. Then the recovered sera were aliquoted in fresh microtubes and kept at − 80 °C until the day of analysis. Serum concentrations of β-carotene and retinol were measured in two occasions (Fig. 1) using high performance liquid chromatography with the method described elsewhere [26].

### Anthropometry

Height and weight were measured using standard procedures [27]. Weight was measured with minimal clothing and without shoes using a digital scale (Soehnle brand) ± 100 g. Height was measured using a non-elastic measuring tape (STABILA brand) while standing against a wall, bare-footed with the scapula in normal circumstance to the nearest 1 cm. Body mass index (BMI) was calculated through dividing weight (kg) by height in meters squared (m^2^).

### Main outcomes

Outcome measures included mean energy and nutrient intake values obtained from the DFFQs and 24-h-recalls.

### Statistical analysis

Demographic characteristics of the study population were tabulated. Normality of distribution was evaluated using the Kolmogorov–Smirnov test. Firstly, de-attenuated correlation coefficients was calculated using the approach described by Rosner [28], to take into account within-person variations caused by day-to-day fluctuations, through following formula: R_true_ = r_observed√ (1 + λ_x_/n_x_) (1 + λ_y_/n_y_) [28], in which λx represents the ratio of the within-and between-person variances for x, and n_x_ represents the number of replicates for the x variable. For this study, n_x_ = 2. Within-and between-person variations (λ_x_) were obtained from its respective intra-class correlations for nutrient intakes estimated by the two FFQs and, n_y_ = 6. Within-and between-person variations (λ_y_) were obtained from its respective intra-class correlations for nutrient intakes estimated by the six 24-h recalls. Reproducibility was measured by Intra-class Correlation Coefficient (ICC).

In the second step, since total energy intake can introduce extraneous variation in recorded food intake, intake estimates were adjusted for total energy intake using the residual method [29].

Finally the degree of agreement based on quartile categorization of nutrients intakes according to the FFQs and the 24-h recalls was evaluated by examining the proportion of subjects classified by the reference method and DFFQs that fell into the same, adjacent, or extreme quartiles.

### Results

Characteristics of study participants are presented in Additional file 2.

Findings of correlation coefficient between DFFQs and 24-h recalls indicate that, compared with recalls, the DFFQs significantly overestimated energy and nutrients intakes (Table 1).

**Table 1:** Degree of association and level of agreement between average daily nutrient intakes by 24-h recalls/biomarkers and Dish-based food frequency questionnaires (DFFQs) (n = 161)

Energy and nutrients (unit)	Average daily intake					Percent agreements			Correlation coefficient between recalls/biomarkers and DFFQs			
	Recall(s)		DFFQ(s)			Same quartile (%)^b^	(same and adjacent quartile)^c^	Opposite quartiles (%)^d^	Unadjusted	Energy-Adjusted^e^	De-attenuated^f^	P. value^g^
	Mean	SD	Mean	SD	P. value^a^				Spearman’ r	Spearman’ r	Spearman’ r	
Energy (kcal/d)	1952.3	59.7	3397.4	1673.4	0.0001	41.2	78	3.1	0.49	–	0.53	0.0001
Protein (g/d)	62.8	19.4	106.4	51.9	0.0001	37.7	82.3	1.8	0.51	0.51	0.54	0.0001
Fat (g/d)	63.3	23.7	107.2	58.9	0.0001	37.5	82.5	6.2	0.45	0.35	0.48	0.0001
Carbohydrate (g/d)	288.4	92.5	324.9	193.6	0.23	33.5	78.4	5.6	0.36	0.17	0.39	0.0001
Dietary fiber (g/d)	13.5	5.6	46.7	28.4	0.0001	34.8	67.7	5	0.30	0.33	0.32	0.0001
Cholesterol (mg/d)	188.7	106.4	324.9	193.6	0.0001	37.3	75.2	5	0.40	0.29	0.41	0.0001
Thiamin (mg/d)	1.9	0.84	4.8	2.9	0.0001	33.9	82.6	2.5	0.50	0.51	0.54	0.0001
Riboflavin (mg/d)	9.5	116.9	2.1	1	0.0001	34.8	77.3	5.8	0.31	0.26	0.32	0.0001
Vitamin C (mg/day)	100.3	50.4	559.2	379.2	0.0001	33.5	70.8	8.6	0.24	0.24	0.26	0.002
Vitamin E (mg/day)	3.9	4.6	8.1	8.8	0.0001	30.3	69	5.8	0.23	0.21	0.24	0.004
Folate (μg/day)	217.1	146.2	438	199.1	0.0001	28.1	68.1	10.6	0.12	0.091	0.13	0.1
Calcium (mg/day)	623.8	259.5	1170.2	550.6	0.0001	41.8	76.1	3.7	0.43	0.41	0.46	0.0001
Iron (mg/day)	17.2	8.8	45.9	27.8	0.0001	33.5	80.3	3.1	0.44	0.42	0.47	0.0001
Zinc (mg/day)	10.8	11.1	43.8	58	0.0001	36.8	71.5	7.6	0.24	0.16	0.25	0.003
Biomarkers												
Retinol (mcg/day)	–	–	–	–	–	–	–	–	0.26	–	0.58	0.0001
Beta-carotene (mcg/day)	–	–	–	–	–	–	–	–	0.13	–	0.40	0.0001

Only data of participants who had two FFQs and a minimum of three 24-h recalls were analyzed

^a^Paired *t* test comparing DFFQ and Recalls in daily average intakes

^b^Percentage of subjects in the same quartile of nutrient intakes from DFFQ1 and 6-day recalls

^c^Percentage of subjects in the same and adjacent quartiles of nutrient intakes from DFFQ1 and 6-day recalls

^d^Opposite (lowest/highest) quartiles

^e^Calculated based on log-transformed value of average daily consumptions with adjustment for total energy intake using the residual method

^f^Correction were calculated based on energy-unadjusted correlation

^g^For all de-attenuated correlations, P < 0.001, except for Folate

The unadjusted Spearman’s correlations for nutrient intakes ranged from 0.091 for folate to 0.51 for protein and thiamin. Energy adjustments slightly improved the correlations for some nutrients including dietary fiber and thiamin.

The de-attenuation correction improved the correlation coefficients for energy, macronutrients and most of micronutrients, statistically significant (P < 0.001).

The mean correlation coefficients between the FFQ and serum concentrations of retinol and β-carotene were 0.26 (P = 0.0001) and 0.13 (P = 0.047), respectively which was improved by de-attenuation to 0.58 and 0.40, respectively (P < 0.001) (Table 1).

Cross classification analysis of two methods into the same and adjacent quartiles of nutrients intake ranged between 67.7% for dietary fiber to 82.6% for thiamin. On average were correctl classified into same or adjacent quartiles and a mean of 5% were classified in opposite quartile.

In order to evaluate the reproducibility of the DFFQ, energy and nutrients intakes obtained from DFFQ-1 were compared with those from DFFQ-2 ranged between 0.23 (carbohydrate) to 0.76 (energy and thiamin) (Table 2).

**Table 2:** Mean ± (SD), Intraclass Correlation Coefficients (ICC) and Spearman correlation coefficients for energy and 13 nutrients between two DFFQs (n = 161)

Energy and 12 nutrients	FFQ_1 M ± SD	FFQ_2 M ± SD	Spearman’s r	ICC
Energy (kcal/day)	3653.7 ± 1940	3004.8 ± 1094	0.60	0.76
Protein (g/day)	113.6 ± 61	96.3 ± 36.9	0.54	0.68
Fat (g/day)	114.2 ± 66.5	94 ± 37.1	0.60	0.75
Carbohydrate (g/day)	559.8 ± 305.8	458 ± 173.6	0.14	0.23
Dietary fiber (g/day)	30.5 ± 18	26.2 ± 11	0.55	0.74
Cholesterol (mg/day)	332.9 ± 214.4	317.7 ± 163.1	0.56	0.72
Thiamin (mg/day)	3.2 ± 1.9	2.6 ± 1	0.55	0.76
Riboflavin (mg/day)	2.2 ± 1.1	1.8 ± 0.7	0.52	0.71
Vitamin C (mg/day)	357 ± 241.5	327.6 ± 170.7	0.48	0.66
Vitamin E (mg/day)	5.5 ± 7.7	4.1 ± 3	0.42	0.48
Folate (μg/day)	467.2 ± 242	406 ± 162.4	0.51	0.75
Calcium (mg/day)	1250.7 ± 672.9	1051.4 ± 410.3	0.56	0.70
Iron (mg/day)	30.3 ± 18.1	25.4 ± 10.3	0.54	0.69
Zinc (mg/day)	30.5 ± 49	21.7 ± 27.4	0.46	0.42

Adjustment for energy was carried using the residual method

### Discussion

In the present study, validation and reliability of a dish-based semi-quantitative FFQ was evaluated. The inclusion of dishes in the list of FFQ food items may improve accuracy and precision of the collected data in two ways in two ways [3, 22]. First, NCDs are linked to culture-specific cooking methods and ingredients [3, 30–32]. Second, in the FFQs in which dishes are not included people may not report invisible parts of a mixed dish because they are neither engaged in their cooking process nor can see the ingredients of different recipes. Consequently, they cannot remember the consumption of mentioned foods [22].

Generally, FFQs are used for ranking individuals according to food or nutrient intake rather than for estimating absolute amounts of intake. In this regard, we used cross-classification analysis whose results for the majority of nutrients were promising, in which approximately more than 60% of participants were classified in the correct quartiles, which is consistent with other studies [12, 20, 26].

In the current study we found that the DFFQ overestimated the consumption of energy and nutrients compared to the 24-h recalls. The issue of overestimation of nutrient intake by FFQs has been reported in other studies [33]. However, if this overestimation is due to a systematic error it can be modified using a correcting factor.

Unadjusted Spearman’s correlation ranged between 0.23 and 0.52, which indicates a fair to medium correlation [12, 34, 35]. The reported correlations are similar to those obtained in other similar validation studies: According to a study carried out in Chile the values between 0.26 and 0.47 [35]. Another study in Colombia the reported correlations between 0.18 and 0.38 in urban areas and between 0.00 and 0.31 in rural areas [12]. In the current study, energy adjustment increased the correlation coefficients for some nutrients but decreased them for the majority of other nutrients. It is documented when the source of variability of nutrient consumption is related to energy intake, energy adjustment increases values of correlation coefficients, however, it will be decreased when it is due to systematic errors (overestimation and underestimation) [36]. In our study, the lower correlation values found in some nutrients may indicate that the DFFQ, to some extent, systematically overestimated intake of those nutrients. However, error in overestimation is expected in the FFQ. Similar to other studies [12, 15, 37] energy adjustment did not improve the crude correlations in our study. Compared with other studies in Denmark [38], Mexico [39], Canada [37], France [40], Ecuador [41], Valencia [42], and Scotland [43], our DFFQ had stronger correlation for total energy, protein, fat, thiamin, carbohydrate, calcium, iron, cholesterol, riboflavin, dietary fiber, zinc, vitamin C, and vitamin E, after the de-attenuation correction.

Compared to other studies reproducibility of the current DFFQ was reasonably acceptable. In other studies the reported correlation coefficients for reliability ranged from 0.06 (for γ-Tocopherol) to 0.31 (for vitamins A and C) [26], and 0.62 (for protein) to 0.88 (for calcium) [44]. In an article the correlation coefficients for FFQs ranged from 0.4 to 0.8 (2009) [40].

To the best of our knowledge this questionnaire is the first valid and reliable DFFQ in Iran which can be used in various epidemiological studies and settings. It ranks people based on their dietary intake and whereby at risk groups can be screened. Effectiveness of nutritional interventions can be evaluated by it, as well. Further studies will reveal the weaknesses of the DFFQ which in turn help in improving its validity.

### Conclusion

The proposed DFFQ in this study showed a relatively acceptable reproducibility and validity in ranking the participants according to energy and nutrients intakes. Therefore it can be used as a reliable tool in epidemiological studies. The DFFQ can evaluate dietary intake among adults in different settings. It can screen out nutritional risk factors and evaluate the effectiveness of interventions, as well.

## Limitation

Since the source of error between the two instruments of food frequency questionnaire and 24-h recall is common and both of them are prone to recall bias the recommended method for such studies is dietary record [24]. However, as the interviewers needed training and we had the time limit, we skipped doing it.

The generalizability of the DFFQ may be limited due to the fact that the study participants were a convenience sample residing in Tehran. The DFFQ needs to be modified and validated according to various climates and food cultures in the country. Therefore the current DFFQ can be used only in climates and food cultures similar to Tehran. Therefore, for studies undertaken in other climates the DFFQ should be modified accordingly.

## Supplementary information

**Additional file 1: Figure S1.** Sampling process of the study subjects for developing the DFFQ. Sampling process.

**Additional file 2: Table S1.** Characteristics of study participants (n = 230, male = 110). Demographic data.

## Data Availability

The dataset supporting the conclusions of this article can be made available upon request after approval by the authors.

## Abbreviations

NCDs: Non-communicable diseases
R: 24-h dietary recall
FFQ: Food frequency questionnaire
DFFQ: Dish-based food frequency questionnaire
ICC: Intraclass correlation coefficient
BMI: Body mass index
RT: Room temperature
Kg: Kilogram

## References

[1] WHO (2013). Global action plan for the prevention and control of noncommunicable diseases 2013–2020.

[2] Ebrahim S (2008). Chronic diseases and calls to action. Int J Epidemiol.

[3] Kim YO (2008). A study testing the usefulness of a dish-based food-frequency questionnaire developed for epidemiological studies in Korea. Br J Nutr.

[4] Esfahani FH (2010). Reproducibility and relative validity of food group intake in a food frequency questionnaire developed for the Tehran Lipid and Glucose Study. J Epidemiol.

[5] Andersen LF, Bere E, Kolbiornsen (2004). Validity and reproducibility of self-reported intake of fruit and vegetable among 6th graders. Eur J Clin Nutr.

[6] Palacios C (2015). Validation and reproducibility of a semi-quantitative FFQ as a measure of dietary intake in adults from Puerto Rico. Public Health Nutr.

[7] Korkalo L (2019). Parents’ reports of preschoolers’ diets: relative validity of a food frequency questionnaire and dietary patterns. Nutrients.

[8] Vilela S (2019). Evaluation of a short food frequency questionnaire for dietary intake assessment among children. Eur J Clin Nutr.

[9] Al Jawaldeh A, Osman D, Tawfik A, World Health Organization. Food and nutrition surveillance systems: a manual for policy-makers and programme managers. 2014.

[10] Fangupo LJ (2019). Relative validity and reproducibility of a food frequency questionnaire to assess energy intake from minimally processed and ultra-processed foods in young children. Nutrients.

[11] Cade J (2002). Development, validation and utilisation of food-frequency questionnaires—a review. Public Health Nutr.

[12] Dehghan M (2012). Validation of a semi-quantitative food frequency questionnaire for Argentinean adults. PLoS ONE.

[13] Almajwal A (2018). Development of food frequency questionnaire (FFQ) for the assessment of dietary intake among overweight and obese Saudi young children. Nutrire.

[14] Kobayashi T (2010). Development of a food frequency questionnaire to estimate habitual dietary intake in Japanese children. Nutr J.

[15] Xia W (2011). Reproducibility and relative validity of a food frequency questionnaire developed for female adolescents in Suihua, North China. PLoS ONE.

[16] Yum J, Lee S (2016). Development and evaluation of a dish-based semiquantitative food frequency questionnaire for Korean adolescents. Nutr Res Pract.

[17] Malekahmadi M (2016). Development, validity, and reliability of a food frequency questionnaire for antioxidants in elderly Iranian people. J Res Med Sci.

[18] Malekshah AF (2006). Validity and reliability of a new food frequency questionnaire compared to 24 h recalls and biochemical measurements: pilot phase of Golestan cohort study of esophageal cancer. Eur J Clin Nutr.

[19] Mohammadifard N (2011). Validity and reproducibility of a food frequency questionnaire for assessment of fruit and vegetable intake in Iranian adults. J Res Med Sci.

[20] Lin P-I (2017). Validation of a dish-based semiquantitative food questionnaire in rural Bangladesh. Nutrients.

[21] Nematy M (2014). Validity and reproducibility of Iranian food frequency questionnaire. Switz Res Park J..

[22] Keshteli A (2014). A dish-based semi-quantitative food frequency questionnaire for assessment of dietary intakes in epidemiologic studies in Iran: design and development. Int J Prev Med.

[23] Mills VC (2015). Relative validity and reproducibility of a food frequency questionnaire for identifying the dietary patterns of toddlers in New Zealand. J Acad Nutr Diet.

[24] Willett W (2012). Nutritional epidemiology.

[25] Amini, M. et al. Development of an application for a dish-based food frequency questionnaire for Iranian population. Med J Islam Repub Iran; under review. 2019. 10.34171/mjiri.34.129 PMC7787032 33437725

[26] Rodriguez C (2017). Development and validation of a food frequency questionnaire to estimate intake among children and adolescents in Urban Peru. Nutrients.

[27] WHO Multicentre Growth Reference Study Group. WHO child growth standards based on length/height, weight and age. Acta Paediatr. 2006;Suppl 450:76–85. http://www.who.int/childgrowth/training/module_b_measuring_growth.pdf. Accessed 23 Mar 2019. 10.1111/j.1651-2227.2006.tb02378.x 16817681

[28] Willett WC (1985). Reproducibility and validity of a semiquantitative food frequency questionnaire. Am J Epidemiol.

[29] Ahmad Esmaillzadeh LA (2008). Food intake patterns may explain the high prevalence of cardiovascular risk factors among iranian women. J Nutr.

[30] Aung W (2018). Fatty acid profiles of various vegetable oils and the association between the use of palm oil vs. peanut oil and risk factors for non-communicable diseases in Yangon region, Myanmar. Nutrients..

[31] Li D (2015). Omega-3 polyunsaturated fatty acids and non-communicable diseases: meta-analysis based systematic review. Asia Pac J Clin Nutr.

[32] Onyango EM, Onyango BM (2018). The rise of noncommunicable diseases in Kenya: an examination of the time trends and contribution of the changes in diet and physical inactivity. J Epidemiol Glob Health.

[33] Moreno-Rojas R, Romero-Saldaña M, Molina-Recio G (2017). Validation of a food frequency questionnaire for the indigenous Épera-Siapidara people in Ecuador. Nutr Hosp.

[34] Mateos LG (2013). Validación de una encuesta para evaluar el estado nutricional y los estilos de vida en las etapas preconcepcional, embarazo y lactancia. Rev Esp Nutr Comunitaria.

[35] Elorriaga N (2015). Validation of a self-administered FFQ in adults in Argentina, Chile and Uruguay. Public Health Nutr.

[36] Landis J. Richard, Koch Gary G. (1977). The Measurement of Observer Agreement for Categorical Data. Biometrics.

[37] Liu L (2013). Assessing the validity of a self-administered food-frequency questionnaire (FFQ) in the adult population of Newfoundland and Labrador, Canada. Nutr J.

[38] Bjerregaard AA (2018). Relative validity of a web-based food frequency questionnaire for Danish adolescents. Nutr J.

[39] Denova-Gutiérrez E (2016). Validity of a food frequency questionnaire to assess food intake in Mexican adolescent and adult population. Salud Pública de México.

[40] Deschamps V (2009). Reproducibility and relative validity of a food-frequency questionnaire among French adults and adolescents. Eur J Clin Nutr.

[41] Villena-Esponera MP (2017). Validation of a Food Frequency Questionnaire for the indigenous Epera-Siapidara people in Ecuador. Nutr Hosp.

[42] Vioque J (2016). Reproducibility and validity of a food frequency questionnaire designed to assess diet in children aged 4-5 years. PLoS ONE.

[43] Hollis JL (2017). Assessing the relative validity of the Scottish Collaborative Group FFQ for measuring dietary intake in adults. Public Health Nutr.

[44] Silva-Jaramillo KM, Neutzling MB, Drehmer M (2015). FFQ for the adult population of the capital of Ecuador (FFQ-Quito): development, reliability and validity. Public Health Nutr.

