# Correction to: Validity and reliability of a dish-based semi-quantitative food frequency questionnaire for assessment of energy and nutrient intake among Iranian adults

**DOI:** 10.1186/s13104-020-05079-1

**Published:** 2020-05-14

**Authors:** Azam Doustmohammadian, Maryam Amini, Ahmad Esmaillzadeh, Nasrin Omidvar, Mitra Abtahi, Monireh Dadkhah-Piraghaj, Bahareh Nikooyeh, Tirang R. Neyestani

**Affiliations:** 1 Department of Nutrition Research, Faculty of Nutrition and Food Technology, National Nutrition and Food Technology Research Institute, Shahid Beheshti University of Medical Sciences, No. 7, Hafezi St., Farahzadi Blvd., P.O.Box: 19395-4741, Tehran, 1981619573 Iran; 2 Department of Community Nutrition, School of Nutritional Sciences and Dietetics, Tehran University of Medical Sciences, Tehran, Iran; 3 Department of Community Nutrition, Faculty of Nutrition and Food Technology, National Nutrition and Food Technology Research Institute, Shahid Beheshti University of Medical Sciences, Tehran, Iran

## Correction to: BMC Res Notes (2020) 13:95 10.1186/s13104-020-04944-3

Unfortunately, the authors’ final corrections only reached us following the publication of the original article [1].

The below have now been updated in the original article:

1. The word ‘and’ has been removed from the last sentence of the first paragraph of the ‘Introduction’.
2. FFQs has been amended to DFFQs in the last paragraph of the ‘Statistical analysis’.
3. FFQ has been amended to DFFQs in the fifth paragraph of the ‘Results’.
4. ‘On average were correctl classified…’ has been amended to ‘On average 73% were correctly classified…’ in the sixth paragraph of the ‘Results’.
5. The duplicate ‘in two ways’ has been removed from the first paragraph of the ‘Discussion’.
6. The fourth paragraph of the ‘Discussion’ has been corrected to read: “…The reported correlations are similar to those obtained in other similar validation studies. According to a study carried out in Chile the values ranged between 0.26 and 0.47 [35]. In another study in Colombia reported correlations ranged between 0.18 and 0.38 in urban areas and between 0.00 and 0.31 in rural areas [12]…”
7. The title Dr. has been added to the names of Hasan Ein-Zinab, Azadeh Nikoosaleh and Hamid Rasekhi in the ‘Acknowledgements’ section.
8. Reference 25 has been corrected to read: Amini M, et al. Development of a dish-based semi-quantitative food frequency questionnaire for Iranian population. Med J Islam Repub Iran. in press **(accepted 18 Aug 2019)**.
