# Supplementary material for: Validity and reliability of a dish-based semi-quantitative food frequency questionnaire for assessment of energy and nutrient intake among Iranian adults
Source: BMC Res Notes. 2020 Feb 24;13:95. doi: 10.1186/s13104-020-04944-3 (PMC7038538; doi:10.1186/s13104-020-04944-3)
Supplement: Supplementary file 1 — Additional file 1: Figure S1. Sampling process of the study subjects for developing the DFFQ. Sampling process. [file 13104_2020_4944_MOESM1_ESM.docx]

51 participants who were under 18 years old were excluded

N=233

Total number of the participants who initiated the study

N=284

12 participants were excluded because of smoking, being on a diet, not having enough number of recalls

N=161

60 participants who did not have the second DFFQ were excluded

N=173

Final number of the participants

N=161

**Additional Figure-** Sampling process of the study subjects for developing the DFFQ
