# Supplementary material for: Validity and reliability of a dish-based semi-quantitative food frequency questionnaire for assessment of energy and nutrient intake among Iranian adults
Source: BMC Res Notes. 2020 Feb 24;13:95. doi: 10.1186/s13104-020-04944-3 (PMC7038538; doi:10.1186/s13104-020-04944-3)
Supplement: Supplementary file 2 — Additional file 2: Table S1. Characteristics of study participants (n = 230, male = 110). Demographic data. [file 13104_2020_4944_MOESM2_ESM.docx]

**Additional Table-Characteristics of study participants (n=230, male=110)**

| **Age group (year)** | **Number (percent)** | |
| --- | --- | --- |
|  | **Male** | **Female** |
| **≤ 32** | 33(30.0) | 18(15.0) |
| **>32-42** | 22(20.0) | 36(30.0) |
| **>42-50** | 22 (20.0) | 41(34.2) |
| **>50** | 33(30.0) | 25(20.8) |
| **Education** | **Number (percent)** | |
|  | **Male** | **Female** |
| **Reading and writing** | 1(0.9) | 1(0.8) |
| **Elementary school** | 9(8.2) | 10(8.3) |
| **Junior high school** | 17 (15.5) | 12 (10.0) |
| **Diploma** | 50 (45.5) | 59 (49.2) |
| **University** | 33 (30.0) | 38(31.7) |
| **Occupation** | **Number (percent)** | |
|  | **Male** | **Female** |
| **Driver** | 57(51.8) | 29 (24.2) |
| **Employee/labor** | 26 (23.6) | 18(15.0) |
| **Housewife** | 0 | 51(42.5) |
| **Student** | 10(9.1) | 12(10.0) |
| **Others** | 15(13.6) | 10(8.3) |
